# Characterization of Particle Shape with an Improved 3D Light Scattering Sensor (3D-LSS) for Aerosols

**DOI:** 10.3390/s24030955

**Published:** 2024-02-01

**Authors:** Marc Weirich, Dzmitry Misiulia, Sergiy Antonyuk

**Affiliations:** Institute of Particle Process Engineering, University of Kaiserslautern-Landau (RPTU), Gottlieb-Daimler-Strasse 44, 67663 Kaiserslautern, Germany; dzmitry.misiulia@mv.rptu.de (D.M.); sergiy.antonyuk@mv.rptu.de (S.A.)

**Keywords:** aerosol, elastic light scattering, online measurement, particle shape

## Abstract

To characterize fine particulate products in industrial gas–solid processes, insights into the particle properties are accessible via various measurement techniques. For micron particles, online imaging techniques offer a fast and reliable assessment of their size and shape. However, for the shape analysis of submicron particles, only offline techniques, such as SEM and TEM imaging, are available. In this work, an online sensor system based on the principle of elastic light scattering of particles in the gas phase is developed to measure the shape factor of non-spherical particles in the size range of 500 nm to 5 µm. Single aerosol particles are guided through a monochromatic circularly polarized laser light beam by an aerodynamic focusing nozzle, which was developed based on the CFD simulation of the flow and particle movement. The intensity of the scattered light is measured at several discrete positions in the azimuthal direction around the particles. An algorithm computes the sphericity of the particles based on the distribution of the intensity signals. The sensor construction, data processing and analysis are described. Model aerosols with particles of different shapes are investigated to test the developed sensor and show its performance in the determination of the sphericity distribution of particles.

## 1. Introduction

Gas phase processes are becoming increasingly important for the synthesis of various fine, dispersed particulate materials, such as catalysts, high-quality pigments or organic electronic components. The product quality is defined by particle properties that can be divided into two categories. One is material properties, such as density or melting point. On the other hand, morphological properties, such as size, shape or specific surface of particles, can significantly influence the behavior of particulate systems [1,2].

While particle size measurements have been a standard tool for quality management and process controls in the industry for years, the determination of particle shape has only gained significance in the past decades [3,4]. The particle shape of fine, dispersed products has been identified as an increasingly important parameter. For example, the particle shape of drug delivery carriers influences the drug release or intake rate [5,6], and the conductivity of battery electric material, as well as the friction and wear behavior of products with carbon black particles, depend significantly on their shape and structure [7].

To control modern processes for the production of particles or aggregates with a specific shape, conventional particle measurement systems which determine a one-dimensional value, e.g., the particle size, specific surface or mobility diameter, are sometimes insufficient. Particle shape can be measured over a wide range of particle sizes via imaging techniques [3]. While modern state-of-the-art systems [8,9] provide inline or online measurements of micron particles, the characterization of submicron particles requires a more extensive, offline procedure. This consist of sampling, sample preparation and examination in an SEM or TEM [10,11] before the process parameters can be adjusted via process control. Although machine learning techniques speed up the microscopic examination step and the developed models provide a statically relevant amount of data, the controlled particle process is still slow to respond to rapid changes in particle generation due to the inevitable time delay caused by the sampling step. This time delay results in rejects or reduced product quality [12,13,14].

To decrease the latency of the process control and to extend available online shape measurement techniques to the submicron particle size range, Dick et al. [15] have shown that the shape of submicron particles can be evaluated via the three-dimensional light scattering profile of single particles crossing a laser beam with a circular polarization.

**Figure 1:**
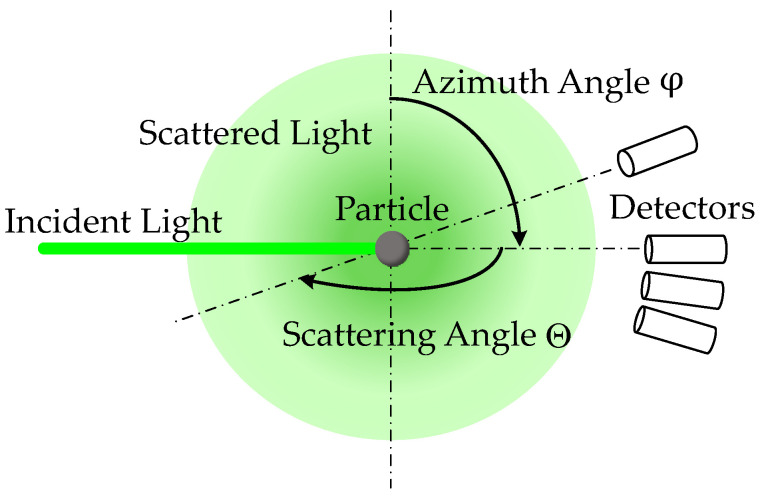
Scattering and azimuth angles around an illuminated particle.

Here, the sphericity is directly linked to the uniformity of the azimuthal light scattering (Figure 1) [16], which shows a homogeneous distribution profile in the case of an ideal sphere.

In our previous work [17] a 3D light scattering sensor (3D-LSS) was developed to determine the particle size in the range of about 500 nm to 5 µm by evaluating the scattering intensity at discrete detector positions. The system was validated via measurements with polystyrene latex (PSL) monospheres with diameters of 1.0 and 2.5 µm. In this work, this sensor system was extended and improved to measure the 3D particle shape by evaluating different detector positions. The developed sensor was validated by measurements with three different test aerosols, namely, spherical PSL, agglomerated SiO_2_ sphere-aggregates of arbitrary shape and cubic NaCl crystals.

## 2. Methodology

### 2.1. Elastic Light Scattering

Light is scattered by inhomogeneities such as particles. If an atom does not absorb an incident light wave, it is set into vibration and emits radiation at the frequency of the incident wave. This physical phenomenon is known as elastic light scattering [18]. Several mathematical models were developed to describe the scattering body around particles by solving the Maxwell equations. Three prominent examples are the Mie theory [19], the van Hulst theory [20] and the discrete dipole approximation (DDA) [21]. Whereas scattering phenomena from Rayleigh, Mie and Fraunhofer regimes are represented, while Mie theory only covers the scattering of spherical particles, van Hulst theory also presents a solution for cylinders. The DDA determines the scattering field of arbitrary bodies, requiring the most computational effort. More details on the elastic light scattering can be found in the literature [20,22,23,24].

### 2.2. Sphericity and Roundness of Particles

Sphericity and roundness are two essential parameters in the effort to describe the shape of particles via measurements of characteristic length sizes, projection areas or the perimeter (2D), as well as the volume and surface area (3D) [25,26,27]. The Krumbein chart [28] was an early tool used to visually characterize the roundness of particles. The underlying principles are still used in commercial particle size analyzers, such as the Morphologi G3SE (Malvern Panalytical Ltd., Cambridge, UK) [29,30].

Wadell [31] defined the so-called true sphericity Ψ as the ratio of the nominal surface area $S_{n}$ (surface area of the volume equivalent sphere) to the surface area of the particle S:

(1) $$\Psi =\frac{S_{n}}{S}=\frac{\sqrt[{3}]{36\pi V²}}{S},$$

where V is the volume of the particle.

The calculation of the Wadell factor requires information about the three-dimensional surface and volume of the particles. For micron particles, these values are usually obtained via expensive and time-consuming microtomography measurements [32,33].

In the two-dimensional plane, the circularity index c is the equivalent of the true sphericity factor.

(2) $$c=\frac{4\pi S_{p}}{P²},$$

where $S_{p}$ represents the projected surface area and P is the projected perimeter. In the case of a perfect sphere, both shape factors are equal to 1 and decrease as the roundness decreases.

### 2.3. Sphericity Index (SPX)

**Figure 2:**
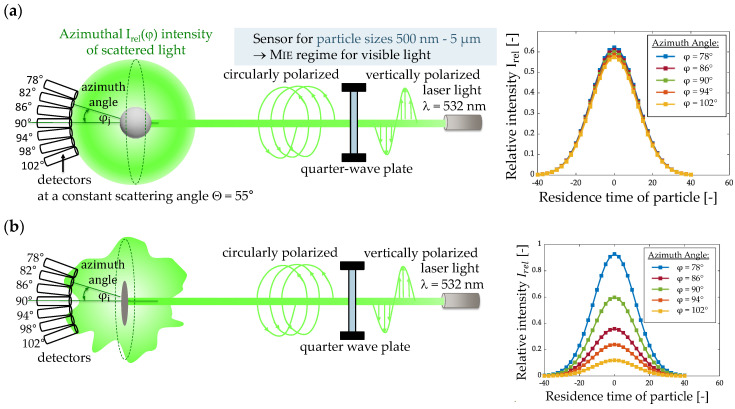
Schematic representation of scattering profiles and relative intensity at different azimuthal positions for a spherical polystyrene (**a**) and non-spherical fiberglass particle (**b**).

The work of Dick et al. [15] showed that the scattering body of a spherical particle illuminated with circularly polarized light has a uniform intensity profile in the azimuthal direction. A non-spherical particle will scatter the light in the azimuthal direction with measurable deviation at different angles, as shown schematically in Figure 2. The measurements of the relative scattering light intensity performed with the developed 3D LSS for spherical polystyrene (Figure 2a) and non-spherical fiberglass dust particles (Figure 2b) demonstrate the influence of the shape on the scattering profile obtained in the angle range of 78° to 102°.

To quantify the deviation of the azimuthal intensity profile from the mean value of relative intensity, the coefficient of variation $Var\left(j\right)$ of all detector signals for each particle j is calculated as

(3) $$Var\left(j\right)=\frac{\sigma (I_{rel,max}(j))}{\bar{I_{rel}}(j)},$$

with the standard deviation of the relative intensity maxima $\sigma (I_{rel,max})$ and the mean value of the relative intensity maxima $\bar{I_{rel}}\left(j\right)$ given as

(4) $$\bar{I_{rel}}\left(j\right)=\frac{1}{K}\sum_{i=1}^{K}I_{rel,max}\left(\varphi_{i},j\right),$$

where K is the number of used detectors at different azimuth angles $\varphi_{i}$, and $I_{max}(\varphi_{i},j)$ describes the maximum intensity measured at detector i for particle j.

A scattered light equivalent shape factor, known as the sphericity index (SPX), describes the shape deviation of a particle from a sphere [15]:

(5) $$SPX\left(j\right)=1-\frac{Var(j)}{\sqrt{K-1}}.$$

### 2.4. Sensor Development and Experimental Setup

**Figure 3:**
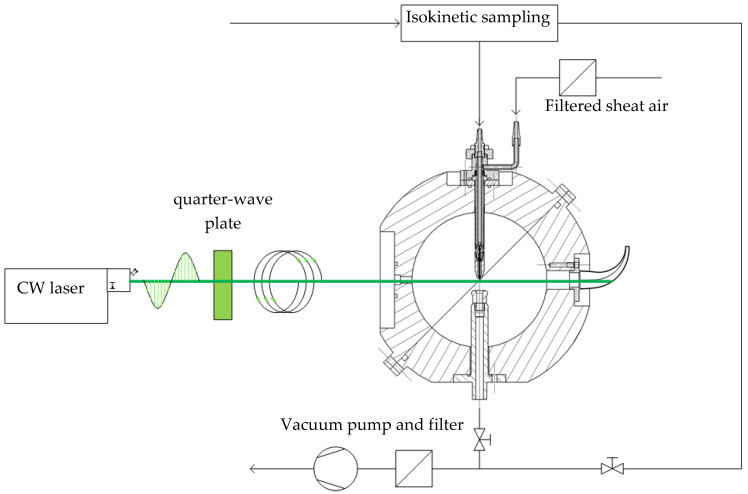
Schematic drawing of the experimental setup and cross-section of the scattering chamber with main components.

The sensor system (Figure 3) consists of a continuous wave (CW) laser (Genesis MX 532 STM, Coherent Inc., Santa Clara, CA, USA) with a wavelength λ of 532 nm emitting vertically polarized coherent light at 500 mW intensity, which is transformed into circularly polarized light by a quarter-wave plate, entering a scattering chamber, housing an aerodynamic focusing nozzle (1) for the sampled particles, an exhaust port (4) with attached vacuum pump, a beam trap (5) and multiple detectors.

To ensure representative sampling, the particles are drawn out of a larger aerosol stream by an isokinetic probe nozzle and transferred to the aerodynamic focusing nozzle (1). This two-fluid nozzle was specially developed (Section 2.4.2) for the alignment and acceleration of fine particles by a sheath air flow (2) into a focused beam of 80 to 100 µm that is orthogonally oriented to the laser beam. The measurement volume (3) is located in the center of the scattering chamber, directly in the cross-section of the laser beam and the aerosol stream. The inner wall surface of the scattering chamber is black anodized to reduce reflections. After exiting the scattering chamber, the laser beam is extinguished in a beam trap (5), while the particles are removed through a suction port (4).

**Figure 4:**
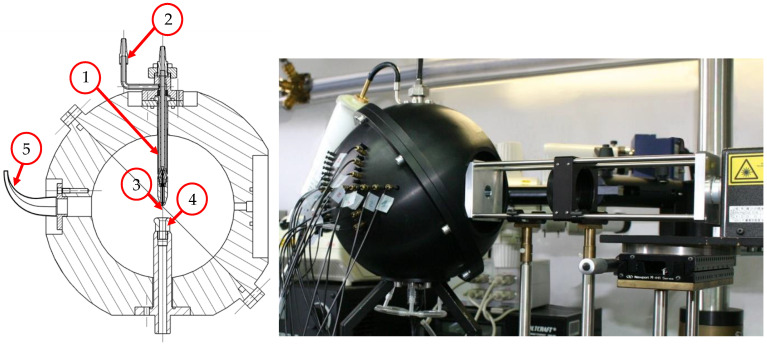
Cross-section of the scattering chamber with main components (**left**) and sensor build (**right**).

The detectors to measure the intensity of the scattered light are placed at seven discrete positions (Figure 4) in the shell at a fixed scattering angle of 55° and azimuth angles between 78° and 102° in 4° steps. The residence time of a particle in the laser beam is evaluated to ensure that only particles crossing the center of the laser beam are considered in the postprocessing. That way, border zone errors or coincidences of multiple particles inside the measuring volume are neglected by deleting signals out of the valid time range, which is estimated by the laser beam’s diameter and the theoretical velocity of the focused particle beam, determined by CFD simulations.

#### 2.4.1. Light Detector Design

**Figure 5:**
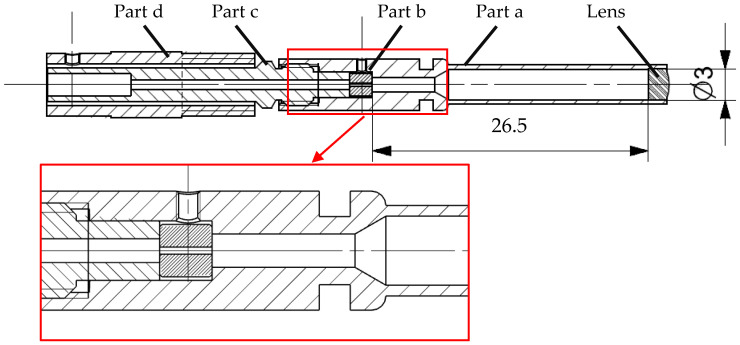
Cross-sectional view of a single light detector.

Since it is only possible to measure the scattered light at a defined surface, rather than at a discrete point, a compromise between the size of the light detection area and the angular resolution is necessary. While a large detection area leads to a stronger and easier detectable signal, the angular resolution will decline. The developed detector setup (Figure 5) consists of an enclosure (Part a) housing a lens and an adjusting collar (Part b) for an optical fiber and tuning pieces (Parts c and d). The lens has a diameter of 3 mm and a back focal length of 26.5 mm. If the inner diameter of the scattering chamber of 140 mm is considered, the angular resolution of the detector is calculated to be about ±1.23°. Due to manufacturing tolerances, the position of the adjusting collar towards the focal point of the lens and the direction of the detector itself towards the center of the scattering chamber need fine-tuning. Therefore, three positioning scrub screws at an angle of 120° are mounted to parts a and d respectively.

After correct alignment, the scattered light received from a particle is coupled to the optical fiber inside the adjusting collar and transferred to a photomultiplier (H10721P-110, Hamamatsu Photonics K.K., Hamamatsu City, Japan), which generates an amplified electric current proportional to the light intensity. The current is consequently transformed into a voltage signal by a transimpedance amplifier and recorded by the data acquisition system (National Instruments, Austin, TX, USA) at a sampling rate of 1 Msps on each channel.

#### 2.4.2. Optimization of the Aerodynamic Focusing Nozzle by CFD Simulations

Another key component of the scattering chamber is the aerodynamic focusing nozzle, which has to guide the particles as close as possible through the center of the measuring volume. In the pioneering work of Roth et al. [34] in the field of aerosol nozzles of single-particle counters, it was shown that the focusing effect of a two-fluid nozzle could be described by

(6) $$\frac{{\dot{V}}_{a}}{{\dot{V}}_{a}+{\dot{V}}_{s}}=\frac{d_{a}^{2}}{d_{0}^{2}},$$

where ${\dot{V}}_{a}\;$ is the sampled aerosol flow rate, ${\dot{V}}_{s}\;$ describes the sheath air flow rate, $d_{0}$ is the outlet diameter of the aerosol nozzle and $d_{a}$ is the diameter of the focused aerosol beam.

Note that the resulting aerosol beam diameter only depends on the gas flow rates and the outlet diameter of the nozzle while the flow inside the nozzle is laminar.

In a previous study [17], a flawed reference design with limitations towards adjustability and robustness was created. To overcome issues during operation, a new nozzle design is developed and evaluated via CFD methods.

**Figure 6:**
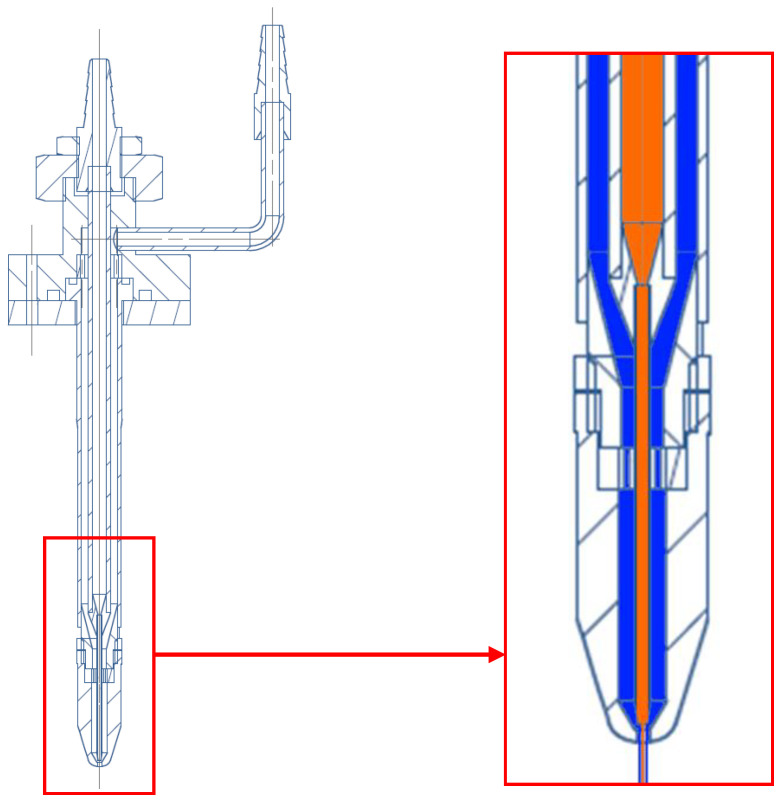
Cross-sectional view of the aerodynamic focusing nozzle and schematics of the sheath air flow (blue) and the aerosol flow (orange).

The new geometry of the aerosol focusing nozzle (Figure 6) is characterized by an aerosol tube with an inner diameter of 1.5 mm and a wall thickness of 0.25 mm, as well as an outlet orifice diameter of $d_{0}=$ 1 mm. The internal flow and the focused aerosol beam are illustrated in the schematic drawing with blue and orange fillings.

**Figure 7:**
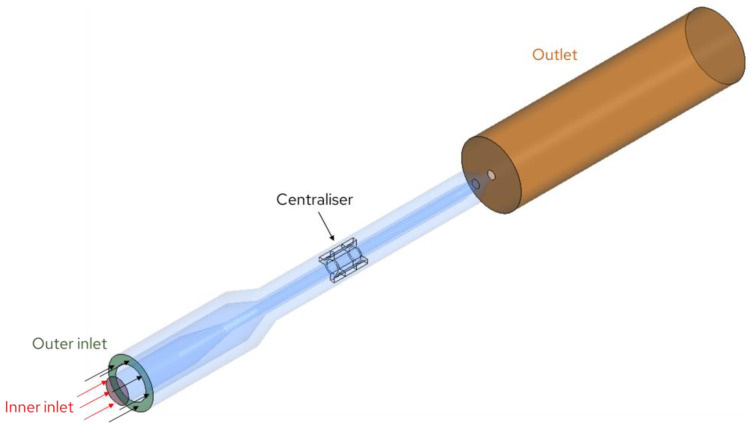
Boundary conditions for CFD simulation of the aerodynamic focusing nozzle.

With a targeted sheath air flow rate ${\dot{V}}_{s}$ of 1.25 cm^3^/min and an aerosol flow rate ${\dot{V}}_{a}\;$ of 10 and 5 cm^3^/min, the diameter of the focused aerosol beam is calculated to be 89 or 63 µm, respectively, via Equation (6). The aerosol tube is centred by a centralizer (Figure 7) with manufacturing tolerances of less than 40 µm in total.

An Euler–Lagrange method has been applied to simulate the gas–solids flow in the nozzle. The gas (air) flow was treated in an Eulerian manner, where the flow variables are a function of space and time and thus are represented as fields. Since the focusing nozzle operates at very low Mach numbers (below 0.2), the gas flow was simulated as an incompressible isothermal flow by the usage of the shear stress transport (SST) k-ω turbulence model [35]. The air properties were taken at a temperature of 20 °C and atmospheric pressure.

The dispersed phase was treated in a Lagrangian fashion, where individual particles are considered, and the position and velocity of each particle are a function of time only. Particle tracking was carried out by forming a set of ordinary differential equations in time for each particle, consisting of equations for position and velocity. Since particle concentration was very low, the effect of the particulate phase on the continuous phase was neglected. Since the particle density is much higher than the density of air, only the drag force, the gravity force and the buoyancy force were considered. The added mass force, the pressure gradient force, the Basset force and lift forces due to the particle rotation (Magnus force) and due to shear in the fluid flow field (Saffman force) are discarded as they have a negligible effect on the particle transport. The effect of flow turbulence on particle tracking was considered by applying the model of turbulent dispersion of particles [36]. More information regarding particle transport modeling settings, as well as the equations for determination drag, gravitational and buoyant forces, can be found in [37].

The following boundary conditions were applied. At the outer inlet, a flow rate of 1.25 dm^3^/min was specified. At the inner inlet, where 10,000 spherical particles, 0.5 and 5 μm in size with a density of 1050 kg/m^3^, were injected with a zero-slip velocity, aerosol flow rates ${\dot{V}}_{a}\;$ of 5 cm^3^/min and 10 cm^3^/min were investigated. The atmospheric pressure was specified at the outlet, and the walls were simulated as non-slip smooth walls.

**Figure 8:**
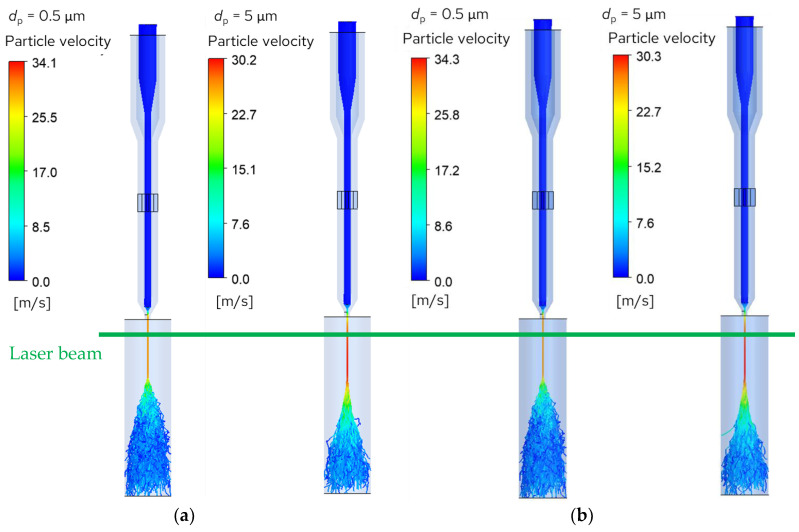
Particle trajectories and velocities in the developed nozzle at flow rates 5 cm^3^/min (**a**) and 10 cm^3^/min (**b**).

The simulated particle trajectories (Figure 8) show that for particles in the size range of 0.5–5 microns and at flow rates of 5 and 10 cm^3^/min, the improved nozzle provides a stable jet, which breaks up sufficiently far downstream of the nozzle, which allows for proper laser scattering.

The aerosol flow rate showed no influence on the maximum particle velocity in the range of 30 to 34 m/s, nor the break-up length of 10 to 12 mm, while the smaller particles reached higher velocities and shorter break-up lengths than the larger particles due to their lower inertia. The aerosol beam diameter $d_{a}$ reaches about 80 µm at 5 cm^3^/min and 100 µm at 10 cm^3^/min aerosol flow. The particle size showed no relevant influence. Compared to the theoretical aerosol beam diameter of 63 (89) µm by the empirical Equation (1), a relative deviation of 27% and 12%, respectively, is found. The larger aerosol beam diameter might be explained by the turbulent flow at the outlet of the nozzle causing a mixing zone between sheath air and aerosol flow; the zero-slip boundary condition at the walls inside the geometry conserving particle paths close to the walls, which are usually extinguished by particle deposition; or the uniform particle distribution at the aerosol inlet, overrepresenting particles closer to the edge of the beam.

### 2.5. Signal Analysis

Once a particle crosses the laser beam, it scatters light towards the shell of the scattering chamber and thus towards the detectors. As explained in Section 2.3, the received light signals are guided from the light detectors to the photomultipliers by optical fibers. In the photomultipliers, the light is converted to electrical currents, amplified and converted to voltage signals and finally digitalized by the data acquisition system.

#### 2.5.1. Sampling Rate

To determine a suitable sampling rate for the digitalization of the analog scatter signal, the Nyquist–Shannon theorem has to be considered [38,39]. The minimal sampling rate of an analog signal is given by the Nyquist rate $f_{sampling}$:

(7) $$f_{sampling}=2\cdot f_{0},$$

where $f_{0}$ is the bandwidth of the analog signal.

With a maximal expected particle velocity of approximately 34 m/s and a 1/e² diameter of 330 µm of the laser beam with a Gaussian intensity distribution, the bandwidth of the analog signal results in $f_{0}=$ 51.5 kHz. It has become good practice to choose a sampling rate at least 2 to 4 times higher than the Nyquist rate [40]. Therefore, the sampling rate of each channel of the sensor system is set to 1 MHz or, respectively, 1 Msps.

#### 2.5.2. Residence Time Analysis

**Figure 9:**
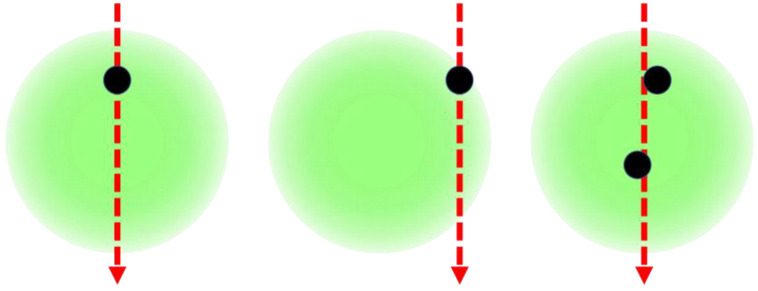
Flight path of a particle with valid signal (**left**), border zone error (**middle**) and coincidence error (**right**) through the laser beam.

The majority of measurement errors in single-particle techniques are caused by border zone and coincidence errors (Figure 9). Border zone errors occur when particles fail to cross the center of the laser beam; while coincidence errors are caused by the simultaneous presence of multiple light scattering particles inside the cylindrical measurement volume of Ø100 × 330 µm. To eliminate those errors, the duration of each particle signal is evaluated.

With an estimated particle velocity from the CFD simulations in the range of 30 to 34 m/s and a beam diameter of 330 µm, the predicted residence time via the movement through the center of the measurement volume is between 9.7 and 11 µs.

**Figure 10:**
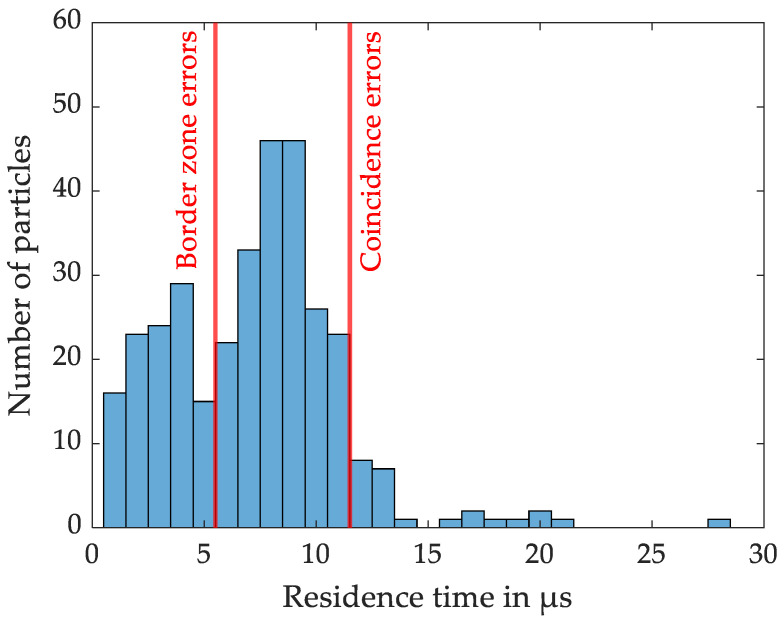
Exemplary residence time analysis of a measurement with model NaCl particles.

In the practical application (Figure 10), here shown with a model aerosol of NaCl particles of approximately 1 µm diameter, the upper time limit was set to 11.5 µs and the lower time limit was set to 5.5 µs. Due to alignment issues between aerosol and laser beam and the detection threshold of the evaluation algorithm, the residence time is arguably slightly shorter in the experimental measurements compared to the simulation.

**Figure 11:**
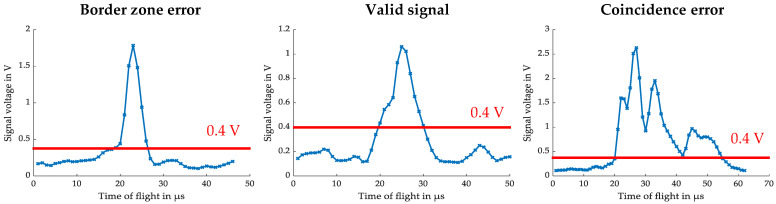
Exemplary signals—Border zone error, valid signal and coincidence error.

Exemplary raw signals for each error category of Figure 10 are presented (Figure 11). Those signals must be filtered. The residence time is determined by the time passed between exceedance and the undercut of a defined threshold of only one detector channel—here, 0.4 V. If a signal is defined as valid on this channel, it is also marked as valid on the other detector channels. The valid signals of all detector channels are stored together with the particle ID and passed to a peak detection algorithm.

Subsequently the detected peaks or maxima are normalized by the maximum value of the same particle id of all channels, resulting in maxima values between zero and one, which are necessary to calculate the sphericity of each particle.

## 3. Materials and Methods

### 3.1. Generation of the Test Aerosols

In this section, the generation of test aerosols, consisting of polystyrene latex monospheres (PSL), cubic sodium chloride crystals and SiO_2_ agglomerates of arbitrary shapes, is described. The materials listed in Table 1 were used.

#### 3.1.1. PSL Aerosol

**Figure 12:**
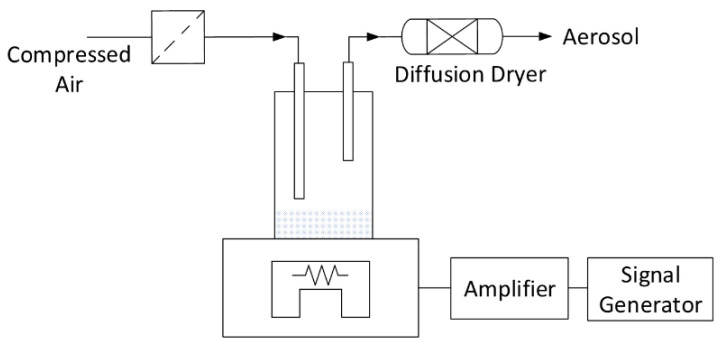
Generator stage to create PSL aerosol.

The practically spherical PSL particles were dispersed in the gas stream within a vibrating dry bed aerosol generator (Wazau GmbH, Berlin, Germany) (Figure 12). The apparatus consists of an inlet for filtered air, a dry bed with a vibration stage underneath and a diffusion dryer filled with silica gel at the outlet. During the experiment, particles are transferred from the fluidized dry bed to the gas stream flowing through the generator stage. The residual moisture is removed by the downstream diffusion dryer.

#### 3.1.2. SiO_2_ and NaCl Aerosol

**Figure 13:**
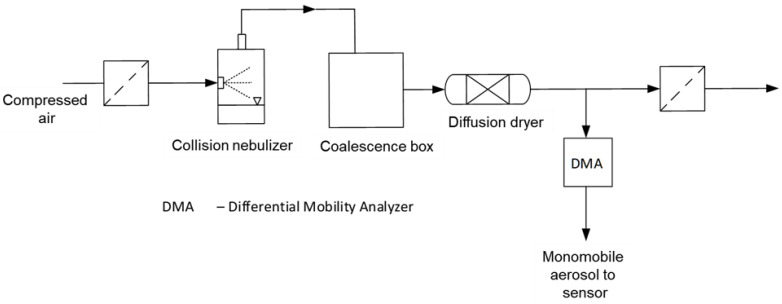
Experimental setup to create a test aerosol consisting of sodium chloride crystals or silica agglomerates.

In a self-made Collison nebulizer [41], a sodium chloride solution (10 Ma-%) and a SiO_2_ suspension were transferred in the gaseous phase (Figure 13). The setup consists of an inlet for filtered air, the nebulizer, a coalescence box, a diffusion dryer filled with silica gel and a differential mobility analyzer (DMA) (Palas GmbH, Karlsruhe, Germany). By increasing the residence time inside the coalescence box, larger agglomerates are formed. The resulting aerosol is dried by the diffusion dryer before entering the DMA. The aerosol is classified based on its electrical mobility, allowing only particles within a narrow range of mobility to exit through the output slit. This monomobile aerosol is guided to the previously described sensor.

### 3.2. Characterization of the Test Aerosols by Scanning Electron Microscopy (SEM) Imaging

**Figure 14:**
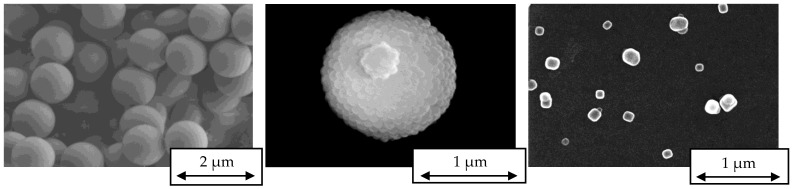
SEM images of PSL monospheres (**left**), silica agglomerate (**middle**) and cubic sodium chloride crystals (**right**).

The generated aerosols were sampled on an electrically conducting substrate with a Nano-Aerosol-Sampler (NAS) (Type 3089, TSI GmbH, Aachen, Germany) for further examination with an SEM. Images of the sampled aerosol are presented in Figure 14.

**Figure 15:**
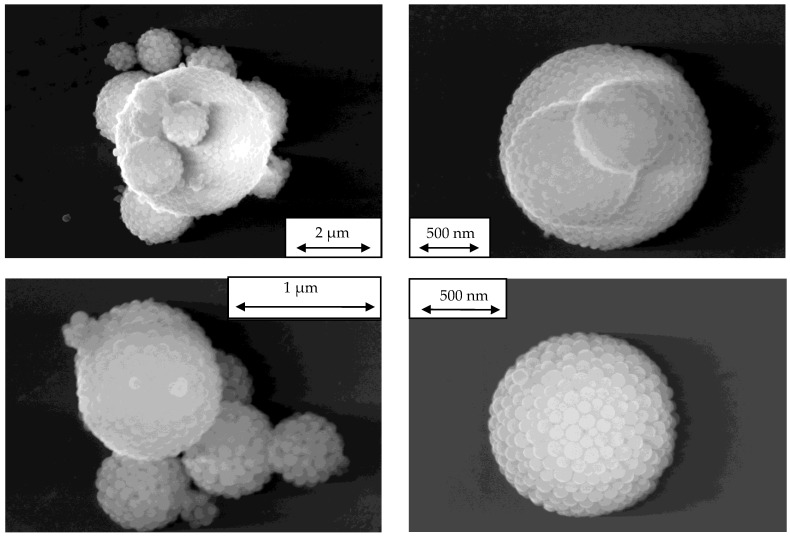
SEM images of the arbitrary shape of silica agglomerates.

While the PSL particles and sodium chloride crystals have a more or less uniform shape, the SiO_2_ agglomerates are present in various shapes (Figure 15). While the Collison nebulizer forms droplets with a given size distribution, the agglomeration process inside the coalescence box is characterized by its stochastic nature due to the varying residence times and the random collision chances of the drying droplets. This leads to the non-uniformity of the formed silica aerosol.

## 4. Results and Discussion

The previously described test aerosols (NaCl and SiO_2_) were classified by a differential mobility analyzer (DMA) to create aerosols with equivalent mobility diameter d_m_ (Table 2). The DMA assumes spherical particles when setting the electrical mobility diameter, which is defined as the equivalent diameter of a sphere with the same mobility in the electrical field as the real non-spherical particle. An overview of the functional principle of a DMA and the electrical mobility diameter is given by [42]. After classification, the test aerosols are transferred to the sensor setup (Section 2).

**Figure 16:**
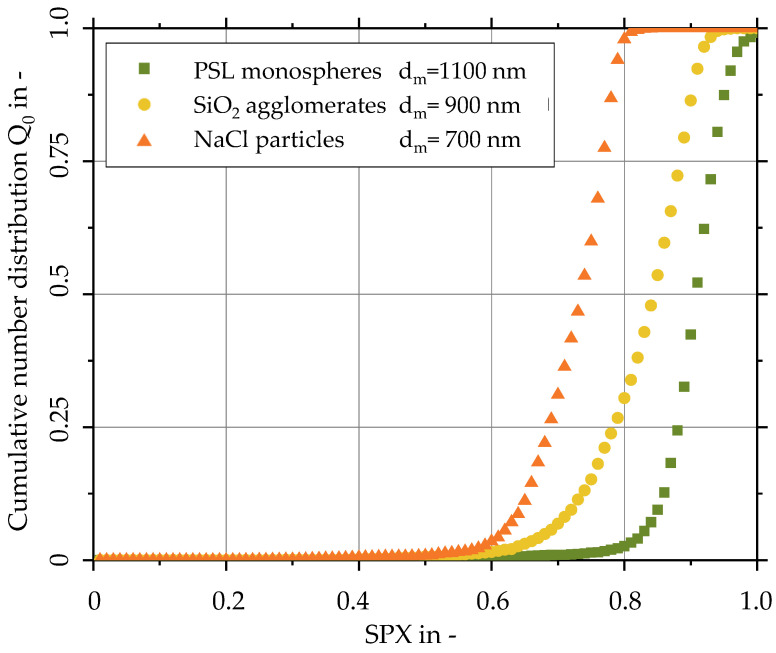
Distributions of the sphericity index (SPX) of polystyrene latex (PSL), silica and NaCl test aerosols measured with the developed 3D-LSS.

The measured intensity profiles of the test aerosols (Figure 16) are evaluated according to Equation (5). The obtained data reveal that the deviation of the particle shape from the spherical shape is observable via the measured SPX values. The cubic sodium chloride crystals show the smallest mean SPX value of all tested aerosols, with a median value of about 0.7. The SiO_2_ agglomerates have a median SPX value of about 0.85, while the PSL monospheres are characterized by the highest mean sphericity index of over 0.9. These SPX values of cube-shaped NaCl particles with defined edge length can be compared with other shape factors obtained via SEM image processing. The mean Wadell factor Ψ in Equation (1) is calculated to be about 0.81, and the mean circularity index c according to Equation (2) results in about 0.79. Assuming a higher sphericity for the NaCl particles (Figure 14) compared to a geometric cube, the Wadell factor for the NaCl particle system should be slightly higher than these estimated values. Taking this into account, the measured SPX values differentiate quite significantly from the conventional definition of sphericity. This deviation is understandable since the presented measurement technique is based on light scattering rather than imaging methods.

The deviations in the measurements for fairly spherical PSL particles from the spherical shape (SPX below 1) might be explained by a systematic error in the calibration step or a noisy data record in the detector setup.

Furthermore, the uniformity of the test aerosols is directly measured by the slope of the distribution curve, where the PSL aerosol shows the highest slope and, consequently, the highest uniformity. The multiple different shapes of the SiO_2_ aerosol, which were already identified in the previous chapter via SEM images, were also detected by the sensor system.

## 5. Conclusions

In this work a 3D light scattering sensor (3D-LSS) was developed to measure the scattered light equivalent shape factor of aerosols in the transitional region between micron and submicron particle sizes (500 nm to 5 µm). The sensor evaluates the scattered light of particles illuminated with circularly polarized light at discrete positions in the azimuthal direction inside a spherical scattering chamber. The intensity profile is analyzed to calculate the sphericity index (SPX) distribution of particles in the aerosol.

An aerodynamic focusing nozzle was designed using CFD simulations to guarantee single-particle flow through the laser beam in the field of view of the light detectors. The particle jet is estimated to have a diameter of 80 to 100 µm and a collimation length of more than 11 mm, providing enough space inside the scattering chamber to guide the laser beam underneath the aerodynamic focusing nozzle. To reduce the impact of border zone errors caused by particles crossing the edge of the laser beam and coincidence errors, due to multiple particles inside the measurement volume, the measurement signal is filtered by a residence time analysis with a valid time range between 5.5 and 11.5 µs.

The differences in the particle shape of the three investigated aerosols were determined by measuring the scattered light profile at seven azimuthal angles between 78° and 102° and a fixed scattering angle of 55° and applying SPX analysis. The polystyrene (PSL) particles have the highest sphericity, followed by NaCl particles and SiO_2_ agglomerates with a higher deviation sphericity. These results are qualitatively supported by SEM images.

In addition, the slope in the cumulative distribution of the SPX increases with the uniformity of the shape of aerosol particles. The PSL particles are highly uniform, whereas the SiO_2_ agglomerates show the greatest shape variation.

In future work, we will investigate how to describe and reduce measurement uncertainties and the size dependency of the SPX. Furthermore, the evaluation algorithms of the presented methods will be directly implemented in an online measurement system in order to reduce the amount of raw data recorded, thus allowing for continuous monitoring of aerosol particle shape.

## Figures and Tables

**Table 1:** Materials used for the experiments.

Material	Purity	Supplier	Size
NaCl	≥99.5%	Carl Roth GmbH, Karlsruhe, Germany	n.a.
SiO_2_	n.a.	microParticles GmbH, Berlin, Germany	119 ± 4 nm
PSL	n.a.	Palas GmbH, Karlsruhe, Germany	1100 nm (narrow distribution)
DI-water	<0.1 µS/cm	RO system + mixed bed desalination	n.a.
Silica gel	n.a.	Carl Roth GmbH, Karlsruhe, Germany	2.5–6 mm

**Table 2:** Setpoints of mobility diameters for the classification step by a DMA.

Test Aerosol	Mobility Diameter d_m_ Setpoint in nm
NaCl	700
SiO_2_	900
PSL	1100

## Data Availability

Data are contained within the article.

## References

[1] Singh P., Ramakrishnan P. (1996). Powder Characterization by Particle Shape Assessment. KONA Powder Part. J..

[2] Hintz W., Antonyuk S., Schubert W., Ebenau B., Haack A., Tomas J., Tsotsas E., Mujumdar A. (2008). Determination of Physical Properties of Fine Particles, Nanoparticles and Particle Beds. Modern Drying Technology.

[3] Ulusoy U. (2023). A Review of Particle Shape Effects on Material Properties for Various Engineering Applications: From Macro to Nanoscale. Minerals.

[4] Polarz S. (2011). Shape Matters: Anisotropy of the Morphology of Inorganic Colloidal Particles-Synthesis and Function. Adv. Funct. Mater..

[5] Champion J.A., Katare Y.K., Mitragotri S. (2007). Particle shape: A new design parameter for micro- and nanoscale drug delivery carriers. J. Control. Release Off. J. Control. Release Soc..

[6] Kho K., Hadinoto K. (2013). Dry powder inhaler delivery of amorphous drug nanoparticles: Effects of the lactose carrier particle shape and size. Powder Technol..

[7] Donnet J.-B., Bansal R.C., Wang M.-J. (1993). Carbon Black: Science and Technology.

[8] Sympatec GmbH Dynamic Image Analysis in Process Environment for Dry Powders, Granules, Suspensions and Emulsion from 1 µm to 10,000 µm. https://www.sympatec.com/en/particle-measurement/sensors/dynamic-image-analysis/pictos/.

[9] Wirz D., Hofmann M., Lorenz H., Bart H.-J., Seidel-Morgenstern A., Temmel E. (2020). A Novel Shadowgraphic Inline Measurement Technique for Image-Based Crystal Size Distribution Analysis. Crystals.

[10] Mills O.P., Rose W.I. (2010). Shape and surface area measurements using scanning electron microscope stereo-pair images of volcanic ash particles. Geosphere.

[11] Babick F. (2016). Characterisation of Colloidal Suspensions. Suspensions of Colloidal Particles and Aggregates.

[12] Bals J., Epple M. (2023). Deep learning for automated size and shape analysis of nanoparticles in scanning electron microscopy. RSC Adv..

[13] Misra S., Li H., He J. (2020). Machine Learning for Subsurface Characterization.

[14] Monchot P., Coquelin L., Guerroudj K., Feltin N., Delvallée A., Crouzier L., Fischer N. (2021). Deep Learning Based Instance Segmentation of Titanium Dioxide Particles in the Form of Agglomerates in Scanning Electron Microscopy. Nanomaterials.

[15] Dick W.D., Ziemann P.J., Huang P.-F., Mcmurry P.H. (1998). Optical shape fraction measurements of submicrometre laboratory and atmospheric aerosols. Meas. Sci. Technol..

[16] Sachweh B.A., Dick W.D., Mcmurry P.H. (1995). Distinguishing between Spherical and Nonspherical Particles by Measuring the Variability in Azimuthal Light Scattering. Aerosol Sci. Technol..

[17] Pitz M., Hellmann A., Ripperger S., Antonyuk S. (2018). Development of a 3D Light Scattering Sensor for Online Characterization of Aerosol Particles. Part. Part. Syst. Charact..

[18] Wriedt T. (1998). A Review of Elastic Light Scattering Theories. Part. Part. Syst. Charact..

[19] Mie G. (1908). Beiträge zur Optik trüber Medien, speziell kolloidaler Metallösungen. Ann. Phys. Chem..

[20] van De Hulst H.C. (2018). Light Scattering by Small Particles.

[21] Purcell E.M., Pennypacker C.R. (1973). Scattering and Absorption of Light by Nonspherical Dielectric Grains. Astrophys. J..

[22] Bohren C.F., Huffman D.R. (2008). Absorption and Scattering of Light by Small Particles.

[23] Mishchenko M.I., Travis L.D., Lacis A. (2002). Scattering, Absorption, and Emission of Light by Small Particles.

[24] Gallinet B., Butet J., Martin O.J.F. (2015). Numerical methods for nanophotonics: Standard problems and future challenges. Laser Photonics Rev..

[25] Cruz-Matías I., Ayala D., Hiller D., Gutsch S., Zacharias M., Estradé S., Peiró F. (2019). Sphericity and roundness computation for particles using the extreme vertices model. J. Comput. Sci..

[26] Blott S.J., Pye K. (2008). Particle shape: A review and new methods of characterization and classification. Sedimentology.

[27] Rodriguez J.M., Edeskär T., Knutsson S. (2013). Particle Shape Quantities and Measurement Techniques–A Review. Electron. J. Geotech. Eng..

[28] Krumbein W.C. (1941). Measurement and Geological Significance of Shape and Roundness of Sedimentary Particles. SEPM J. Sediment. Res..

[29] Gresina F., Farkas B., Fábián S.Á., Szalai Z., Varga G. (2023). Morphological analysis of mineral grains from different sedimentary environments using automated static image analysis. Sediment. Geol..

[30] Szmańda J.B., Witkowski K. (2021). Morphometric Parameters of Krumbein Grain Shape Charts—A Critical Approach in Light of the Automatic Grain Shape Image Analysis. Minerals.

[31] Wadell H. (1933). Sphericity and Roundness of Rock Particles. J. Geol..

[32] Zhou B., Wang J., Zhao B. (2015). Micromorphology characterization and reconstruction of sand particles using micro X-ray tomography and spherical harmonics. Eng. Geol..

[33] Lin C.L., Miller J.D. (2005). 3D characterization and analysis of particle shape using X-ray microtomography (XMT). Powder Technol..

[34] Roth C., Gebhart J., Heigwer G. (1976). Spectrometry of submicron-aerosols by counting single particles illuminated by laser light. J. Colloid Interface Sci..

[35] Menter F.R. (1994). Two-equation eddy-viscosity turbulence models for engineering applications. AIAA J..

[36] Gosman A.D., Loannides E. (1983). Aspects of Computer Simulation of Liquid-Fueled Combustors. J. Energy.

[37] Misiulia D., Andersson A.G., Lundström T.S. (2017). Effects of the inlet angle on the collection efficiency of a cyclone with helical-roof inlet. Powder Technol..

[38] Nyquist H. (1928). Certain Topics in Telegraph Transmission Theory. Trans. Am. Inst. Electr. Eng..

[39] Shannon C.E. (1949). Communication in the Presence of Noise. Proc. IRE.

[40] Das S., Mohanty N., Singh A. Is the Nyquist Rate Enough?. Proceedings of the 2008 the Third International Conference on Digital Telecommunications (ICDT 2008).

[41] Kerner M., Schmidt K., Schumacher S., Puderbach V., Asbach C., Antonyuk S. (2020). Evaluation of electrostatic properties of electret filters for aerosol deposition. Sep. Purif. Technol..

[42] Gensdarmes F. (2015). Methods of Detection and Characterization. Nanoengineering.

